# Optimisation of custom-made footwear in people with diabetes at high ulcer risk: biomechanical evaluation of the DIASSIST footwear intervention

**DOI:** 10.1080/19424280.2025.2490679

**Published:** 2025-06-20

**Authors:** Lisa E. Vossen, Sicco A. Bus, Jaap J. Van Netten

**Affiliations:** a Department of Rehabilitation Medicine, Amsterdam UMC, University of Amsterdam, Amsterdam, The Netherlands; b Amsterdam Movement Sciences, Rehabilitation & Development, Amsterdam, The Netherlands

**Keywords:** Plantar pressure, footwear intervention, ulcer, diabetes, biomechanics

## Introduction

Custom-made footwear helps prevent foot ulcer recurrence in people with diabetes by redistributing pressure and offload high risk regions of the foot. Its effectiveness is evaluated using in-shoe plantar pressure measurements. Based on these measurements, modifications can be made to existing footwear to improve offloading. Ten years ago, the DIAFOS trial was conducted to investigate the effect of footwear design and modifications on pressure reduction (Arts et al., 2015). Based on these results, a state-of-the-art design protocol for custom-made footwear was published to provide guidelines for footwear design (Bus et al., 2020). Key in this protocol is to achieve peak pressures below 200 kPa.

## Purpose of the study

As part of the DIASSIST randomised controlled trial (RCT) (Vossen et al., 2023), we aimed to investigate if plantar pressure from custom-made footwear in daily practice can be optimised, including investigating if pressures below 200 kPa can be further reduced.

## Methods

Of 126 participants of the DIASSIST RCT, 62 were assigned to the intervention arm and included in this cohort analysis. All participants had diabetes, peripheral neuropathy, a recently healed foot ulcer or (partial) foot amputation and were in possession of custom-made footwear. A total 102 pairs of footwear were assessed on design and measured during walking using in-shoe plantar pressure measurements (Pedar-X). Modifications were made to the footwear if necessary based on the design assessment and pressure measurements. Footwear was modified until the research team assessed that further modifications would not be beneficial. Multiple peak plantar pressure parameters (Vossen et al., 2025), were calculated pre- and post-modifications, and changes in plantar pressure were evaluated using paired t-tests and statistical parametric mapping. Additionally, we assessed the number and type of modifications made. Subgroup analyses were performed on shoes <200 kPa pre-modifications.

## Results

**Figure 1.**
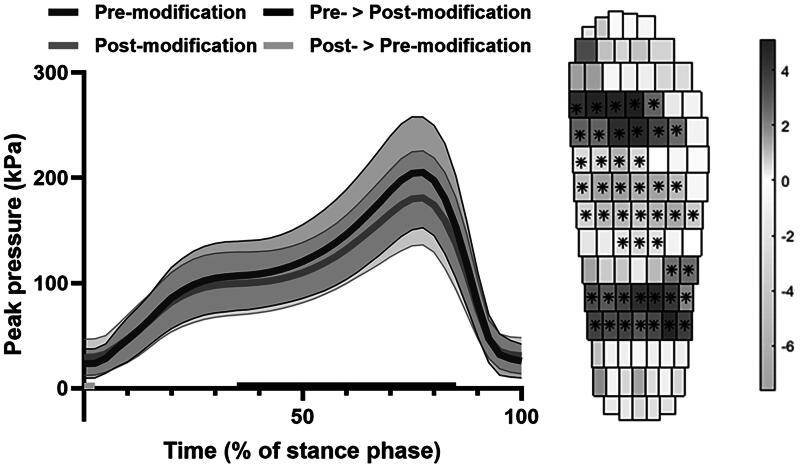
Peak plantar pressure in temporal (left) and spatial (right) parameters. Spatial results are shown as the statistical differences, with significant differences indicated with an asterisk. Positive values indicate higher values pre-, negative values post-modifications.

A total of 201 shoes were analysed. Modifications were made to 122 shoes, of which 47 (39%) were <200 kPa pre-modifications. The most applied modifications were the addition (n = 47) or adaptation (n = 23) of a transmetatarsal bar to the insole, and local deepening and/or padding of areas in the insole (n = 61). Plantar pressure was significantly reduced post-modifications for the scalar parameters (Table 1) and multidimensional parameters in the forefoot area and from 35% to 85% of the stance phase (Figure 1). For footwear <200 kPa pre-modifications, significant improvements were found for the scalar parameters (Table 1), but not for the multidimensional parameters.

**Table 1.** Scalar peak plantar pressure parameters pre- and post-modifications of all modified shoes and shoes <200 kPa pre-modifications.

	Analysis	Pre-modifications	Post-modifications	Mean difference	P
PMax (kPa)	All (n = 101)	226 (56)	199 (48)	27 (37)	<0.001
	<200 kPa (n = 37)	173 (21)	164 (20)	10 (25)	<0.025
PTI **(**kPa**.s)**	All (n = 101)	103 (30)	93 (23)	10 (21)	<0.001
	<200 kPa (n = 37)	87 (19)	82 (16)	5 (13)	<0.05
PGrad (kPa/mm)	All (n = 101)	16 (6)	13 (5)	3 (4)	<0.001
	<200 kPa (n = 37)	11 (3)	10 (3)	1 (3)	<0.01

PMax = maximum peak pressure, PTI = pressure time integral, PGrad = pressure gradient. Data are presented as mean (SD).

## Discussion and conclusion

Significant improvements to custom-made footwear were made with the DIASSIST intervention. This shows that plantar pressure of custom-made footwear in daily clinical practice can be reduced, even when pressures are already below 200 kPa Clinical outcomes of the full DIASSIST RCT are needed to determine its effects on ulcer recurrence.
